# Longitudinal Claudin Gene Expression Analyses in Canine Mammary Tissues and Thereof Derived Primary Cultures and Cell Lines

**DOI:** 10.3390/ijms17101655

**Published:** 2016-09-29

**Authors:** Susanne C. Hammer, Annegret Becker, Katja Rateitschak, Annika Mohr, Florenza Lüder Ripoli, Silvia Hennecke, Johannes Junginger, Marion Hewicker-Trautwein, Bertram Brenig, Anaclet Ngezahayo, Ingo Nolte, Hugo Murua Escobar

**Affiliations:** 1 Small Animal Clinic, University of Veterinary Medicine Hannover, Bünteweg 9, 30559 Hannover, Germany; schammer@tiho-hannover.de (S.C.H.); annika.mohr@tiho-hannover.de (A.M.); florenza@ripoli.com.br (F.L.R.); hugo.murua.escobar@med.uni-rostock.de (H.M.E.); 2 Division of Medicine, Haematology, Oncology and Palliative Medicine, University of Rostock, Ernst-Heydemann-Str. 6, 18055 Rostock, Germany; 3 Institute of Biophysics, Leibniz University Hannover, Herrenhäuser Straße 2, 30419 Hannover, Germany; a.becker@biophysik.uni-hannover.de (A.B.); ngezahayo@biophysik.uni-hannover.de (A.N.); 4 Institute for Bioinformatics, University Medicine Greifswald, Walther-Rathenau-Str. 48, 17475 Greifswald, Germany; katja.rateitschak@uni-greifswald.de; 5 Institute of Veterinary Medicine, Georg-August-University Göttingen, Burckhardtweg 2, 37077 Göttingen, Germany; shennec@gwdg.de (S.H.); bbrenig@gwdg.de (B.B.); 6 Department of Pathology, University of Veterinary Medicine Hannover, Bünteweg 17, 30559 Hannover, Germany; johannes.junginger@tiho-hannover.de (J.J.); marion.hewicker-trautwein@tiho-hannover.de (M.H.-T.); 7 Center for Systems Neuroscience (ZSN) Hannover, University of Veterinary Medicine Hannover, Bünteweg 17, 30559 Hannover, Germany

**Keywords:** claudin, mammary neoplasias, canine, cell lines, cell culture, marker expression

## Abstract

Human and canine mammary tumours show partial claudin expression deregulations. Further, claudins have been used for directed therapeutic approaches. However, the development of claudin targeting approaches requires stable claudin expressing cell lines. This study reports the establishment and characterisation of canine mammary tissue derived cell lines, analysing longitudinally the claudin-1, -3, -4 and -7 expressions in original tissue samples, primary cultures and developed cell lines. Primary cultures were derived from 17 canine mammary tissues: healthy, lobular hyperplasia, simple adenoma, complex adenoma, simple tubular carcinoma, complex carcinoma, carcinoma arising in a benign mixed tumour and benign mixed tissue. Cultivation was performed, if possible, until passage 30. Claudin mRNA and protein expressions were analysed by PCR, QuantiGene Plex Assay, immunocytochemistry and immunofluorescence. Further, cytokeratin expression was analysed immunocytochemically. Cultivation resulted in 11 established cell lines, eight showing epithelial character. In five of the early passages the *claudin* expressions decreased compared to the original tissues. In general, claudin expressions were diminished during cultivation. Three cell lines kept longitudinally claudin, as well as epithelial marker expressions, representing valuable tools for the development of claudin targeted anti-tumour therapies.

## 1. Introduction

The claudin (CLDN) protein family is a major structural and functional component of the tight junction complex in epithelial and endothelial cells [1,2,3]. Changes in the CLDN expressions were found in human and canine epithelial neoplasms of the mammary gland [4,5,6], human prostate [7,8], canine pancreas [9,10], colorectum of both species [11,12,13,14,15] and evaluated regarding the diagnostic potential of those proteins. Furthermore, CLDN proteins act as a receptor for the enterotoxin of *Clostridium perfringens* (*Clostridium perfringens* enterotoxin, CPE), a feature that enables a directed CLDN targeting for therapeutic approaches [16,17]. This feature was already used to successfully kill human CLDN-3 and -4 expressing cells in vivo and in vitro [7,16,17,18,19]. Directed recombinant mutation of the CPE sparing the cytotoxic domain leads to a protein that allows a directed reversible binding of CLDN proteins without killing the targeted cell [19,20,21]. This enables drug delivery to CLDN expressing cells [22]. Thus, CLDN targeting has been discussed to represent a new therapeutic option for CLDN expressing tumours, although side effects appearing in CLDN expressing non-neoplastic cells have to be considered. Consequently, a potential application should be carefully chosen, e.g., intratumoural injections for local application [5,7,18,19,23,24,25]. Tumour derived cell lines are a key tool for in vitro evaluation of CLDN targeting as a therapeutic option. Furthermore, understanding the regulation of CLDN expressions in tumour cells increases general insights in tumour biology. In general, cell lines can also serve in comparative cancer genetics and translational medicine, as reported for canine lung tumour derived cell lines, showing genetic and biochemical similarities to human lung tumours and promising effects of a drug effective in human tumours on these cell lines [26].

Several studies indicated that cells derived from human and canine tumours reduce their CLDN expressions during cultivation [4,7,18,27]. Consequently, the CLDN expression profiles of cell lines do not always reflect the expression profiles of the original tissues. Thus CLDN-expressing tumour derived cell lines, keeping the CLDN expressions, are of major value for tumour research.

This study reports comparative CLDN-1, -3, -4 and -7 expression analyses of non-neoplastic and neoplastic canine mammary gland tissue samples and thereof derived primary cultures and cell lines. The aim of the study was to identify stable CLDN expressing canine mammary gland derived cell lines, keeping the initial epithelial character.

## 2. Results

### 2.1. Cell Cultivation and Analyses of the Epithelial Character

#### 2.1.1. Primary Cultures

Six of the 17 primary cultures discontinued growth before reaching p.19: three of the five healthy tissues derived primary cultures (DT14/04R, DT14/05S2, DT14/07R), one of two lobular hyperplasia derived primary cultures (DT14/08R) and both simple carcinoma derived primary cultures (DT14/07T, DT14/09T). For details see Table 1.

For the evaluation of the epithelial character, early and late passages were immunocytochemically analysed for cytokeratin distribution. DT14/04R and DT14/05S2 did not exhibit any cytokeratins. DT14/07T partially kept the distribution of cytokeratins. DT14/08R and DT14/09T lost the cytokeratins. DT14/07R added on cytokeratins. For details see Table 2.

#### 2.1.2. Cell Lines

Eleven of the 17 primary cultures proliferated until p.19 and beyond and therefore resulted in cell lines: two of the five healthy tissue derived primary cultures (DT14/05R, DT14/06R), one of the two lobular hyperplasia derived primary cultures (T124), eight of the twelve neoplastic tissue derived primary cultures (simple adenoma derived (DT14/04T), complex adenoma derived primary cultures (T121), complex carcinoma derived primary cultures (T120A, DT14/06T, DT14/06TS), carcinoma arising in a benign mixed tumour derived primary culture (T126), benign mixed mammary tumours derived primary cultures (DT14/10, DT15/02T). For details see Table 1.

Seven cell lines partially kept the distribution of cytokeratins (T124, T121, T120A, DT14/06T, DT14/06TS, DT14/10, DT15/02T). Three cell lines lost the cytokeratins (DT14/05R, DT14/06R, T126). One cell line added on cytokeratins (DT14/04T). For details see Table 3.

### 2.2. CLDN Gene Expression Analyses

#### 2.2.1. Gene Expression Analyses: QuantiGene Plex Assay

The QuantiGene Plex Assay allowed the simultaneous multiplexing of all genes of interest (*CLDN-1*, *-3*, *-4*, *-7*, *β-Actin* (*ACTB*) and *glyceraldehyde-3-phosphate dehydrogenase* (*GAPDH*)) in RNAs of all examined passages of the primary cultures, cell lines and available original tissue samples via amplified fluorescence detection. Furthermore, all passages of the primary cultures, cell lines and the original tissue samples were analysed in one run. *CLDN* expressions were normalized to the reference genes *ACTB* and *GAPDH* revealing similar results. *ACTB* and *GAPDH* were chosen as they were already used for *CLDN* gene expression analyses in canine prostate tumour and mammary tissue and tumour derived cell lines [27]. Furthermore, they were used for *CLDN* gene expression analyses of human tissues and intestinal carcinomas [13,14,28,29]. If a *CLDN* expression was normalized only to *ACTB* or *GAPDH* it is clearly stated within the text.

##### *CLDN* Gene Expression Alterations from Tissue Samples to Passage 00

Alterations of the *CLDN* expressions presumably result from dissociating cells from the tissue matrix in the process of exposing them to cultivation. These alterations were analysed in three primary cultures and three cell lines by comparing the gene expressions of the original tissue samples and the corresponding first passage (p.00). 

**Figure 1:**
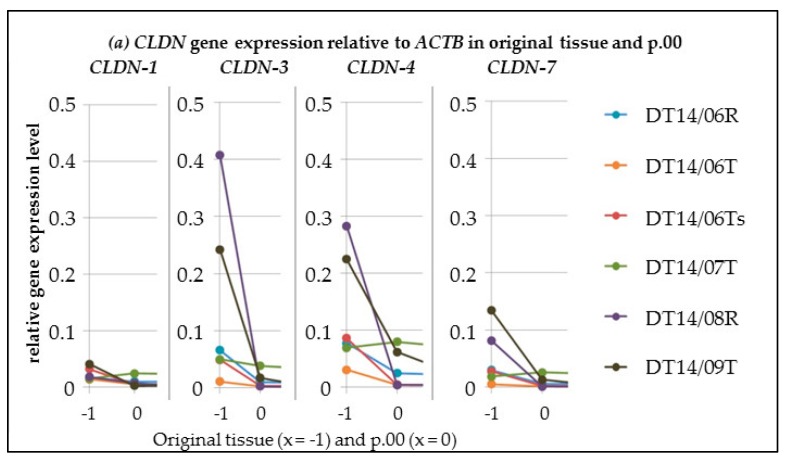

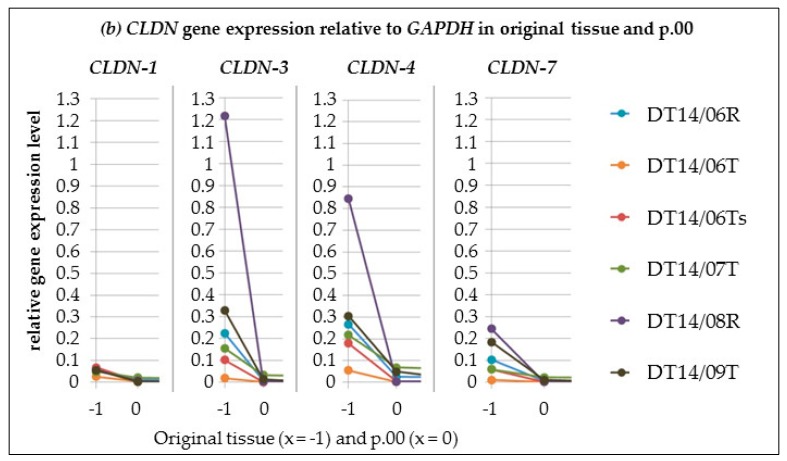
This figure shows the *claudin* (*CLDN*) gene expression profiles from original tissue samples (*x*-axis = −1) to p.00 (*x*-axis = 0): *CLDN-1*, *-4* and *-7* gene expressions in relation to *β-Actin* (*ACTB*) (**a**) increased slightly in culture DT14/07T (green line) and decreased in the other cultures, the same as for the *CLDN-1*, *-4* and -*7* gene expressions in relation to *glyceraldehyde-3-phosphate dehydrogenase* (*GAPDH*) (**b**), and *CLDN-3* gene expression in relation to both reference genes in all cultures.

In all three analysed primary cultures and all three analysed cell lines, the *CLDN-1*, *-3*, *-4* and *-7* gene expressions decreased in relation to *GAPDH,* comparing the original tissue samples and the corresponding first passage. In five of six cell cultures the *CLDN-1*, *-3*, *-4* and *-7* gene expressions decreased in relation to *ACTB*. In one cell culture the *CLDN-1*, *-4* and *-7* gene expressions increased in relation to *ACTB.* However, in that cell culture *CLDN-3* expression decreased in relation to *ACTB.* For details see Figure 1.

##### *CLDN* Gene Expressions in the Latest Analysed Passage

**Figure 2:**
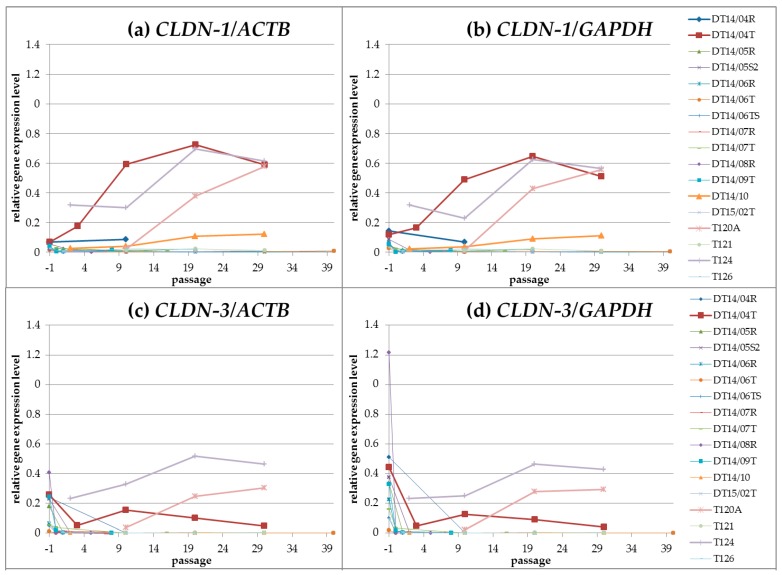

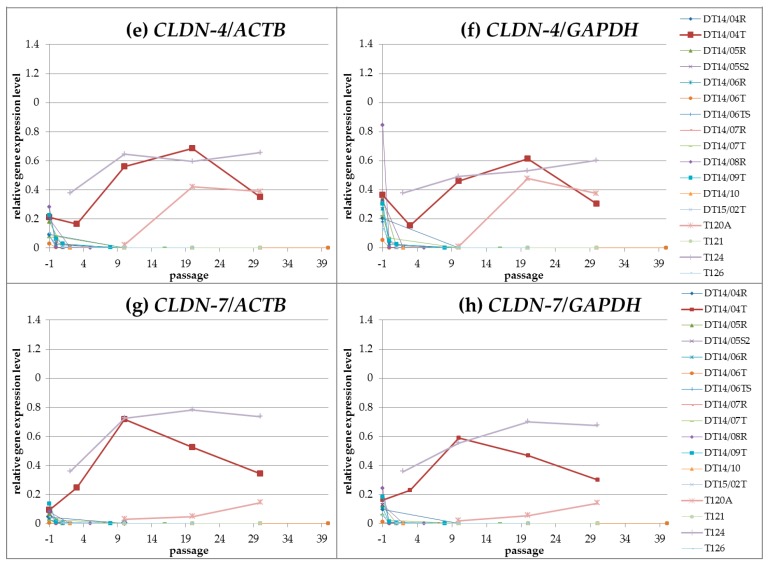
*CLDN* gene expression profiles of canine mammary non-neoplastic and neoplastic tissue derived cell cultures during cultivation until passage 30: Cell lines T120A, T124, DT14/04T and DT14/10 exhibited high *CLDN-1* (**a**,**b**) gene expressions; in the other cell cultures the expression was below 0.1 (*CLDN-1* relative to *ACTB* and *GAPDH* respectively: T120A: 0.58, 0.56; T124: 0.61, 0.56; DT14/04T: 0.59, 0.51; DT14/10: 0.12, 0.11); Cell lines T120A and T124 exhibited high *CLDN-3* (**c**,**d**) gene expressions; in the other cell cultures the expression was below 0.1 (*CLDN-3* relative to *ACTB* and *GAPDH* respectively: T124: 0.47, 0.43; T120A: 0.30, 0.29); Cell lines T124, DT14/04T and T120A exhibited high *CLDN-4* (**e**,**f**) and *-7* (**g**,**h**) gene expressions; in the other cell cultures the expression was below 0.1 (*CLDN-4* relative to *ACTB* and *GAPDH* respectively: T124: 0.66, 0.60; DT14/04T: 0.35, 0.30; T120A: 0.39, 0.38; *CLDN-7* relative to *ACTB* and *GAPDH* respectively: T124: 0.74, 0.68; DT14/04T: 0.34, 0.30; T120A: 0.14, 0.14).

High *CLDN* gene expression levels (cut off was set at level 0.1, referring to level 1 as gene expression of the reference genes *ACTB* and *GAPDH*) in the latest analysed passage were displayed for *CLDN-1* by four cell lines (T124, DT14/04T, T120A and DT14/10), for *CLDN-3* by two cell lines (T124 and T120A) and for the *CLDN*s*-4* and *-7* by three cell lines (T124, DT14/04T and T120A). For details see Figure 2.

##### *CLDN* Gene Expression Profiles during Cultivation (Passages 00 until Passage 30)

The *CLDN-1*, *-3*, *-4* and *-7* gene expression levels decreased in the majority of the analysed primary cultures and cell lines in the beginning of the cultivation and remained low (lower than 0.1) during cultivation. 

Cell lines T120A and T124 showed high *CLDN* expression levels for the *CLDN-1*, *-3*, *-4* and *-7* genes. Cell line DT14/04T exhibited high *CLDN* expression levels for the *CLDN-1*, *-4* and *-7* gene*s*. Therefore, the *CLDN* gene expression profiles were described for cell lines T120A, T124 and DT14/04T.

T120A: Cell line T120A was analysed in p.10, p.20 and p.30. Generally, the *CLDN-1*, *-3* and *-4* expressions were higher than the *CLDN-7* expression. Regarding the expression profiles from p.10 to the latest analysed p.30, the *CLDN-1*, *-3* and *-7* gene expressions increased. The *CLDN-4* expression increased from p.10 to p.20 but decreased slightly from p.20 to p.30.

T124: Cell line T124 was analysed in p.02, p.10, p.20 and p.30. Passage 30 displayed comparable expression levels for the *CLDN-1*, *-3*, *-4* and *-7* genes. Regarding the expression profiles from p.02 to p.30, the *CLDN* gene expressions increased. Regarding the expression profiles from p.20 to p.30 the *CLDN-4* gene expression increased, whereas the *CLDN-1*, *-3* and *-7* gene expressions decreased moderately.

DT14/04T: Cell line DT14/04T was analysed in p.03, p.10, p.20 and p.30. *CLDN-1*, *-3*, *-4* and *-7* expression levels of the latest analysed p.30 were at high levels. However, a decrease of expression was observed from p.10 for *CLDN-3* and *-7* and from p.20 for *CLDN-1* and *-4*.

##### Linear Correlation of *CLDN* Expression during Cultivation

**Figure 3:**
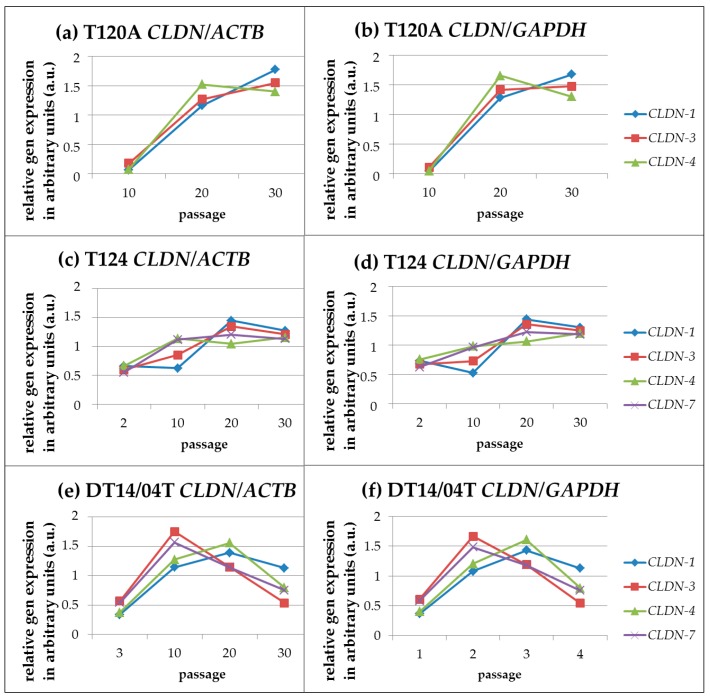
The temporal *CLDN* gene expression profiles during cultivation showed highly correlating *CLDNs* within cell line T120A for the *CLDN-1/-3*, *-1/-4* and *-3/-*4 genes (in relation to *ACTB* (**a**) respectively 0.99; 0.91; 0.96, and in relation to *GAPDH* (**b**) respectively 0.98; 0.90; 0.97***)***, within cell line T124 for the *CLDN-1/-3* and *-4/-7* genes (in relation to ACTB (**c**) respectively 0.94; 0.95, and in relation to GAPDH (**d**) 0.97; 0.93) and within cell line DT14/04T for the *CLDN-1/-4* and *-3/-7* genes (in relation to ACTB (**e**) respectively 0.90; 0.98, and in relation to GAPDH (**f**) 0.90; 0.97).

Analyses of the correlation of *CLDN* gene expressions during cultivation revealed highly correlating *CLDN* gene expression profiles in cell line T120A for the *CLDN-1/-3*, *-1/-4* and *-3/-4* genes, in cell line T124 for the *CLDN-1/-3* and *-4/-7* genes and in cell line DT14/04T for the *CLDN-1/-4* and *-3/-7* genes. For details see Figure 3.

#### 2.2.2. Gene Expression Analyses: Conventional PCR

*CLDN-1, -3, -4* and *-7* gene expressions were analysed using conventional PCR in cDNA of early and late passages of cell cultures T120A, T121, T124, DT14/04R, DT14/04T, DT14/06T and DT14/10 allowing a qualitative statement. Previous to the conventional PCR, contamination with genomic DNA were excluded for all cDNAs using a primer assay for an intro sequence of *C-X-C chemokine receptor type 4* gene.

*CLDN-1* gene expression was equal in four cell lines (T120A, T124, DT14/04T; DT14/10) and one primary culture (DT14/04R). In two cell lines (DT14/06T, T121), *CLDN-1* expression differed from negative in the early to positive in the late passage. *CLDN-3* gene expression was equal in three cell lines (T120A, T124, DT14/04T) and lost in another cell line (DT14/06T). In one primary culture (DT14/04R) *CLDN-3* expression differed from negative in the early to positive in the late passage and in two cell lines (T121, DT14/10) the other way round. *CLDN-4* gene expression was equal in three cell lines (T120A, T124, DT14/04T) and lost in three cell lines (T121, DT14/06T, DT14/10). In one primary culture (DT14/04R) *CLDN-4* expression differed from negative in the early to positive in the late passage. *CLDN-7* gene expression was equal in three cell lines (T120A, T124, DT14/04T) and lost in two cell lines (DT14/10, DT14/06T). In one primary culture *CLDN-7* gene expression differed from negative in the early to positive in the late passage (DT14/04R) and the other way round in one cell line (T121). For details see Table 4.

### 2.3. Immunocytochemistry

Immunocytochemistry detected CLDN-1, -3, -4 and -7 proteins in cell cultures T120A, T124, DT14/04T, DT14/04R, DT14/10, DT14/06T and T121. The late passages of cell cultures DT14/04T and DT14/10 lacked CLDN-4 protein.

### 2.4. Immunofluorescence

**Figure 4:**
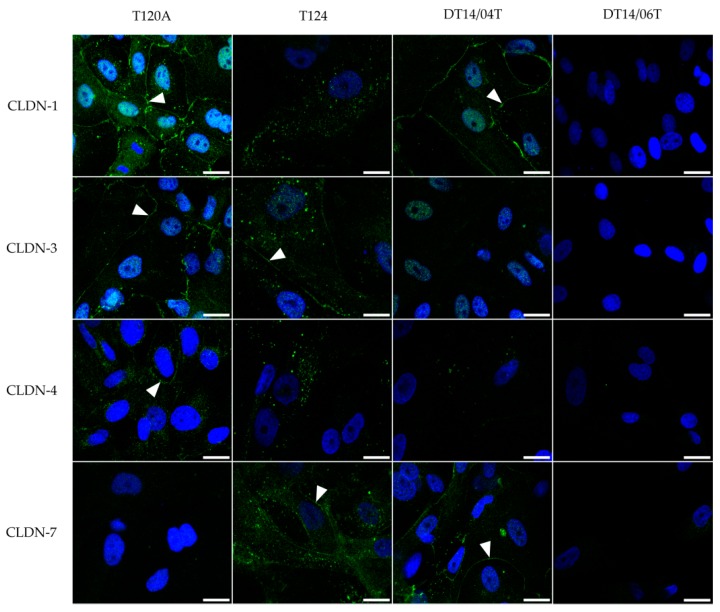
CLDN protein distribution and localization in cell lines T120A (p.37–42), T124 (p.12), DT14/04T (CLDN-1, -3 and -7: p.16-19; CLDN-4: p.5) and DT14/06T (p.44–49) as detected by IF. Cells were fixed and stained for CLDN-1, -3, -4 and -7 using fluorescein or FITC (fluorescein isothiocyanate) conjugated secondary antibodies, which are visible in green, and for nuclei using DAPI (4′,6-diamidino-2-phenylindol), which is visible in blue. Arrows indicate CLDN localization at cell–cell contacts. Scale bar: 20 µm.

Immunofluorescence revealed a cell specific expression of the CLDN-1, -3, -4, and -7 proteins. While cell line DT14/06T p.44–49 did not show an expression of the CLDNs, cell lines T120A, T124 and DT14/04T did. The expression of CLDN-1, -3 and -4 proteins was found in cell line T120A p.37–42 at the cell membrane, but there was no signal for CLDN-7. All four analysed CLDN-1, -3, -4 and -7 proteins were found in cell line T124. In cell line T124 p.15 the CLDN-3 and -7 proteins were localized at the cell membranes, CLDN-1 and -4 were punctually localized in the cytoplasm. In cell line DT14/04T p.16–19 CLDN-1 and -7 proteins were strongly distributed in the cell-to cell contact regions, CLDN-4 was weakly expressed in p.5 and CLDN-3 was not detected in p.16–19. For details see Figure 4.

## 3. Discussion

Mammary tumours commonly occur in female dogs [30]. Treatment options are restricted to invasive options such as surgery and, additionally, chemotherapy in the case of metastasis [31]. Retrospective studies exploring cytotoxic agents are mostly restricted to a limited number of dogs, included in the respective studies. Complex prospectivly conducted studies evaluating experimental therapeutic protocols are practically missing [32]. However, the establishment of non-invasive therapeutic approaches for mammary carcinomas is currently addressed in various ways. A recent study reported a non-invasive treatment option for mammary tumours in dogs and cats using plasmonic photothermal therapy [33]. Due to disease similarities between humans and dogs, similar environmental conditions, but easier performable trials (guidelines are less strict than for humans), dogs are considered to represent an excellent model for human diseases [34]. Accordingly, data obtained by canine tumour research may also lead to progress in human tumor research. Further experimental therapeutic approaches targeting human cancer cells include the use of the enterotoxin of *Clostridium perfringens* (CPE) in order to kill cancer cells by directed CLDN binding. In a xenografical mouse model, the intratumourally injected CPE reduced the size of CLDN expressing tumours initiated by a human breast cancer cell line [5]. A recombinant CPE-tumour necrosis factor fusion protein inhibited growth of CLDN expressing human ovarian tumour cells [22]. Human ovarian tumour cells overexpressing CLDNs-3 and -4, injected into mice, were selectively killed by the administration of CPE [18]. Further, CLDN-3 and -4 expressing cells from a human prostate carcinoma bone marrow metastase were killed in vitro by adding the CPE to the culture medium [7]. Additionally, mutated recombinant CPE fragments are in experimental evaluation as for example the non-toxic but CLDN binding C-terminal fragment (C-CPE), which is able to inhibit the growth of CLDN expressing tumour cells [5,16,22,24]. A combination of the C-CPE and taxol enhanced chemosensitivity of human ovarian cancer cells and inhibited tumour growth of such cells in a xenografic mouse model [24].

In order to develop specific CLDN targeting therapeutic approaches for canine mammary gland tumours, the availability of CLDN expressing cell lines is a precondition. In the present study, comparative CLDN gene and protein expression analyses on eleven cell lines and six primary cultures derived from canine mammary gland non-neoplastic and neoplastic tissues were performed. In the majority of the herein analysed cell lines and primary cultures (fourteen of seventeen) the *CLDN-1*, *-3*, *-4* and *-7* gene expressions decreased after transfer to culture conditions when compared to the original tissue samples. Furthermore, CLDN expressions remained low during longitudinal cultivation over 30 passages. These results match our previously published study, showing low to missing CLDN expression in long term established canine mammary non-neoplastic and neoplastic tissue derived cell lines ZMTH3, MTH53A and MTH52C [27]. Further studies evaluating CLDN expression in different tumour types reported a decrease or lack of CLDN-3, -4 and -7 expressions of in vitro cultured cells compared to uncultured cells [4,7,18]. Human prostate carcinoma metastases derived cell lines PC3 and LNCaP lack CLDN-3 and -4 expressions, whereas human non-neoplastic prostate epithelium expresses the CLDNs-3 and -4 [7]. A 1000-fold lower CLDN-7 expression was found in cultivated human mammary endothelial cells (HMECs) compared to uncultured HMECs [4]. A comparable decrease regarding *CLDN-3* and *-4* gene expression patterns was reported for cultured human ovarian cancer cell lines that show a lower CLDN expression than primary ovarian tumours [18]. The reason for CLDN expression or repression for the tumour cells is not fully understood. It has been proposed that the loss of CLDNs allows the loss of cell polarity, thereby promoting the tumorigenesis [35]. Metastasis is a process linked to advanced tumour stage, in which cells dissociate from a tumour, enter blood or lymphatic vessels, attach in distant sites and form new tumours [36]. Epithelial-to-mesenchymal transition (EMT) is linked to metastasis [37] and it is suggestive that loss of CLDNs contributes to this process [12,29]. However, the role of CLDNs in EMT is not yet elucidated. As in cultured cells, the complex three dimensional structure of tissues is non-existent, the observed down regulation can be caused by the loss of required CLDN functions. 

However, in three of the herein evaluated new established canine mammary cell lines (originating from respectively a lobular hyperplasia, a carcinoma and an adenoma) expression of the *CLDN* genes increased comparing an early passage and p.30, but did not reach constant expression levels before p.30. Further, these cell lines kept the epithelial character throughout the observed cultivation period. To the best of our knowledge, this is the first study to longitudinally analyse the CLDN expressions up to passage 30. Comparisons to other studies are difficult as information about the analysed passages are missing.

Correlation coefficient analyses of the three CLDN expressing cell lines identified in the present study indicated that culturing conditions do not affect all CLDNs in the same manner. These findings might be associated to the functional ability of different CLDNs to specifically heterophilically interact with each other. CLDN-3 is able to interact heterophilically with CLDN-1 or CLDN-2, whereas the latter are not able to interact with each other [38]. Immunofluorescence revealed that CLDN-1 and -4 proteins in cell line T124 were located in the cytoplasm, whereas the CLDN-3 and -7 proteins were located at the cell membranes, where the CLDN proteins are supposed to be located [39]. Such apparent mislocalizations were also described for the CLDN-3 protein in human breast cancer derived cell lines and may be related to local invasiveness [40]. These findings should receive attention in studies evaluating the CLDNs as a target for anti-tumour therapies. 

In summary, the data reported here show that cultivation of canine mammary cells has negative effects on the expression of different CLDNs. However, three epithelial canine mammary lobular hyperplasia, adenoma and carcinoma derived cell lines kept the expression of the analysed CLDN genes and proteins during cultivation until p.30. These three canine mammary gland tissue derived CLDN expressing epithelial cell lines represent valuable tools for further studies regarding CLDN targeted tumour therapeutic approaches.

## 4. Materials and Methods

### 4.1. Material, Cell Cultivation and Sample Processing

#### 4.1.1. Canine Mammary Gland Tissue Samples

Five normal canine mammary gland tissue samples, two lobular hyperplasias, one simple adenoma, one complex adenoma, two simple carcinomas, three complex carcinomas, one carcinoma arising in a benign mixed tumour and two benign mixed mammary tumours were provided by the Small Animal Clinic, University of Veterinary Medicine Hannover, Germany, the Institute of Veterinary Medicine, Georg-August-University Göttingen, Germany, and the veterinary practice of Dr. M. Schilling, Bielefeld, Germany; in total 17 canine mammary gland tissue samples. 

Samples for diagnostic procedures were stored in formalin. Histological classification was performed in the Department of Pathology of the University of Veterinary Medicine Hannover, Germany, according to the proposed classification of canine mammary tumours [41]. Depending on the size of the tissue samples, portions for gene expression analyses were shock frozen in liquid nitrogen and stored at −80 °C until RNA was isolated using the RNeasy^®^ Mini Kit (Qiagen, Hilden, Germany). For details see Table 1.

#### 4.1.2. Establishment of Primary Cultures

Non-neoplastic and neoplastic tissue samples were stored in Hanks Medium (Hank’s salts L201-00, Biochrom GmbH, Berlin, Germany), 2% penicillin and streptomycin (Biochrom GmbH, Berlin, Germany) until being processed. A small portion of the initial tissue sample was chopped into small pieces followed by collagenase treatment (0.35% Collagenase NB 8 Broad Range, SERVA Electrophoresis GmbH, Heidelberg, Germany) at 37 °C until cell connections dissolved, for a maximum of 4 h. The dissociated cells were washed with medium 199 (Gibco by Life technologies^TM^, Darmstadt, Germany) containing 20% fetal calf serum (FBS Superior, Biochrom GmbH, Berlin, Germany) and 200 IU/mL penicillin and 200 mg/mL streptomycin (Biochrom GmbH, Berlin, Germany). Afterwards, cells were transferred into sterile 25 cm^2^ flasks containing 5 mL medium 199 (20%). The cultures were incubated in 5% CO_2_/air at 37 °C. During the first days, the cells were monitored on a daily basis observing cell growth, depending on respective characteristics. The culture medium was changed at least twice per week. Reaching confluency, the cells were passaged by adding 1 mL TrypLE^TM^ Express ([-] Phenol Red, Gibco by Life technologies^TM^, Darmstadt, Germany) and then the cell suspension was split and transferred to sterile cell culture flasks, containing 5 mL medium 199 (20%) per flask.

The cells dissolved from the initial tissue sample were herein indicated as passage (p.) 00 (p.00). After first splitting, the cells were termed p.01, higher numbers indicate the number of the certain passage. The term “primary culture” referred to cell cultures between p.00 and p.19. Reaching p.20 and above, the certain “primary culture” was furthermore designated as “cell line”.

Primary cultures were analysed in p.00 and additionally in another early passage (p.01, p.02). In case cell growth discontinued before the tenth passage, the latest viable passage was analysed. In case cells grew beyond p.10 but discontinued growth before p.19, then p.10, and additionally the latest viable passage, were analysed. 

Cell lines were analysed in p.00, in an early passage (p.01–p.03) and then in p.10, p.20 and p.30.

Depending on the cell number of a passage, pellets were generated according to Table 1 and stored adequately.

#### 4.1.3. Generation of Cell Pellets

The cells were detached by adding 1 mL TrypLE^TM^ Express ([-] Phenol Red, Gibco by Life technologies^TM^, Darmstadt, Germany) to the flasks. The cells were counted using Cellometer SD100 cell counting chambers (Nexcelom Bioscience, Lawrence, MA, USA) and Cellometer^TM^ Auto T4 (Nexcelom Bioscience), pelleted at 1000 *g* for 10 min and stored adequately until further usage.

For histological analyses, pellets were stored in 4% paraformaldehyde. For gene expression analyses, using either QuantiGene Plex Assay (Affymetrix, Santa Clara, CA, USA) or conventional PCR, cell pellets were stored at −80 °C.

#### 4.1.4. Cell Lysis for Gene Expression Analysis Using QuantiGene Plex Assay (Affymetrix)

Cells were lysed at a final concentration of 400 cells/µL Lysis Mixture using the QuantiGene Sample Processing Kit (Affymetrix), according to the manufacturer’s instructions. Lysates were stored at −80 °C until usage.

#### 4.1.5. RNA-Isolation from Tissue Samples for Gene Expression Analyses Using QuantiGene Plex Assay (Affymetrix)

5–30 mg of frozen tissue samples were homogenized using the Tissue Lyser II (Qiagen, Hilden, Germany) and 5 mm Stainless Steel Beads (Qiagen) according to the manufacturer’s instructions (Qiagen). RNA was isolated using the RNeasy^®^ Mini Kit (Qiagen) including a digestion step for genomic DNA using the RNase-Free DNase Set (Qiagen) according to the manufacturer´s instructions. An additional step for digestion of genomic DNA was performed using RQ1-DNAse (Promega, Mannheim, Germany) according to the manufacturer’s instructions. The total amount of RNA was quantified using Synergy 2 (Biotek, Bad Friedrichshall, Germany) controlled by Gen5™ Reader Control and Data Analysis Software. RNAs were stored at −80 °C until usage.

#### 4.1.6. RNA-Isolation from Cultured Cells for Gene Expression Analyses Using Conventional PCR

Cells were homogenised using QIAshredder^TM^ columns (Qiagen). RNA was isolated using the RNeasy^®^ Mini Kit (Qiagen), RNase-Free DNase Set (Qiagen) and RQ1-DNAse (Promega) as described in Section 4.1.5. Measurement of the amount of RNA and storage was performed as described in Section 4.1.5.

### 4.2. Gene Expression Analyses

#### 4.2.1. *CLDN* Gene Expression Analyses Using QuantiGene Plex Assay (Affymetrix)

*CLDN-1*, *-3*, *-4*, *-7*, *β-actin* (*ACTB*) and *glyceraldehyde 3-phosphate dehydrogenase* (*GAPDH*) gene expressions were simultaneously analysed in isolated RNA (12.5 ng/µL) of canine mammary non-neoplastic and neoplastic tissue samples and cell lysates (400 cells/µL) of different passages of thereof derived primary cultures and cell lines using QuantiGene Plex Assay (Affymetrix) according to the manufacturer’s instructions, Vortemp 56 (Labnet International, Inc., Edison, NJ, USA) and Luminex 100/200 Systems (Luminex Corporation, Austin, TX, USA). Luminex 100/200 Systems detected the amplified fluorescence signals generated by the QuantiGene Plex Assay (Affymetrix). For each run, background controls and process controls were performed as duplicates. As process controls, samples that were to be analysed were chosen. Samples were run as unicates. Normalization was performed towards two reference genes, *ACTB* [13] and *GAPDH* [42]. Available tissue samples and passages of the corresponding cell cultures were analysed according to Table 1.

Probes for the QuantiGene Plex Assay (Affymetrix) were ordered based on the mRNA sequences provided by the National Center for Biotechnology Information (NCBI) and custom designed by Affymetrix (Santa Clara, CA, USA). The accession numbers for the mRNA sequences were as follows: *CLDN-1*: XM_845155; *CLDN-3*: NM_001003088; *CLDN-4*: XM_005620962; *CLDN-7*: XM_546584; *ACTB*: XM_536888; *GAPDH*: NM_001003142.

##### *CLDN* Gene Expression in the Original Tissue Samples and Passages 00

*CLDN* expression alterations from original tissue samples to p.00 were analysed in cultures DT14/06R, DT14/06T, DT14/06TS, DT14/07T, DT14/08R and DT14/09T, as RNA from tissue samples and lysates from the first cultivated cells were available.

##### *CLDN* Expression in the Latest Analysed Passage

*CLDN* expression was declared as “high”, if the expression level in the latest analysed passage was higher than cut off 0.1, as “low”, if the expression level was lower than cut off 0.1. Normalization was performed towards the reference genes *ACTB* and *GAPDH*, all values refer to the reference gene value 1.

##### Linear Correlation of *CLDN* Expression during Cultivation

The correlation coefficient according to Pearson is an empirical correlation coefficient and can be applied to calculate linear correlations between two time-dependent gene expression profiles, which were obtained from the cultivation of cell lines T120A, T124 and DT14/04T. The correlation coefficient is defined by the following equation:

$$Kor_{e}(x,y)=\frac{\sum_{i=1}^{n}\left(x_{i}-xm\right)\times \left(y_{i}-ym\right)}{\sqrt{\sum_{i=1}^{n}{\left(x_{i}-xm\right)}^{2}\times \sum_{i=1}^{n}{\left(y_{i}-ym\right)}^{2}}}$$

in which *x* and *y* are passage series for two different *CLDNs* of the same cell line. “*xm*” and “*ym*” are the mean values of the *CLDN* gene expression data of a passage series in a certain cell line. For a better visibility of correlations, each value of each *CLDN* gene expression profile was scaled with a factor. The value of the factor is equal to the mean value of the expression time series such that all scaled expression profiles have the same mean value.

#### 4.2.2. *CLDN* Gene Expression Analyses Using Conventional PCR

##### cDNA-Synthesis

cDNA was synthesised using M-MLV-Reverse Transcriptase (Promega), 500 ng of total RNA and AP2-Primer according to the manufacturer´s instructions. Two negative process controls were included. To check reagents for contamination, a negative control was performed once for each run that included the reverse transcriptase but no RNA, so that no cDNA was synthesisable from sample RNA. To check the RNA for contamination, an additional negative control was performed for each sample and included RNA but no reverse transcriptase, so that cDNA could not be synthesised from the RNA. Genomic DNA contamination was excluded in cDNA and negative controls using a PCR assay (CXCR4 upVIIIa/CXCR4 lo VIII) for an intron sequence of the reference gene *C-X-C chemokine receptor type 4* (*cxcr4*) which can only be found on genomic DNA, but not on cDNA. Integrity of the RNA and thereby cDNA quality of all samples was tested using a PCR assay for the reference gene *ACTB* (ACTB up/ACTB lo). cDNA was further analysed if contaminations were excluded in the cDNA and negative controls and if integrity of RNA was verified. Primer sequences have been published previously [27].

##### *CLDN* Gene Expression Analyses: Conventional PCR

*CLDN-1*, *-3*, *-4* and *-7* gene expressions were analysed in 1 µL cDNA of an early and late passage of cell cultures T120A, T121, T124, DT14/04R, DT14/04T, DT14/06T and DT14/10, using PCR assays (*CLDN-1*: CL_1_up1/CL_1_lo1; *CLDN-3*: CL_3_up1/CL_3_lo1; *CLDN-4*: CL_4_up1/CL_4_lo1; *CLDN-7*: CL_7_up1/CL_7_lo1) and real-time PCR primer assays (*CLDN-1*: CL1 sg up1/CL1 sg lo1; *CLDN-3*: CL3 sg up3/CL3 sg lo3; *CLDN-4*: CL4 sg up1/CL4 sg lo1; *CLDN-7*: CL7 sg up1/CL7 sg lo2) for conventional PCR, as published previously [27]. The standard protocol for conventional PCR contained initial denaturation at 95 °C for 10 min; followed by 35 cycles: denaturation at 95 °C for 30 s, annealing at 60 °C for 30 s and elongation at 72 °C for 1 min for PCR assays or 30 s for real-time PCR assays; followed by the final elongation at 72 °C for 5 min.

### 4.3. Immunocytochemistry

For immunocytochemical characterisation of the cytokeratin distribution in the cell cultures, cell pellets from early and late passages of the cultured cells were detached with TrypLE^TM^ Express Enzyme (Thermo Fischer Scientific, Darmstadt, Germany) followed by mechanical scraping (Cell Scraper M, TPP, Trasadingen, Switzerland) if enzymatic detachment was incomplete. Detached cells were centrifuged at 1000× *g* for 10 min, fixed in 4% paraformaldehyde and embedded in paraffin wax. Pellets of all primary cultures and cell lines were immunocytochemically stained with a panel of antibodies detecting various cytokeratins (CK), for details see Table 2 and Table 3. In addition, pellets of the primary culture DT14/04R and cell lines T120A, T121, T124, DT14/04T, DT14/06T and DT14/10 were further investigated using antibodies specific for the CLDN-1, -3, -4 and -7 proteins according to Table 5. Immunocytochemistry was performed on serial sections according to standard procedures as described previously [43]. Briefly, antigen retrieval was achieved by incubation in a citrate buffer (pH 6.0, 20 min, 95 °C) followed by inhibition of endogenous peroxidase activity with 0.5% H_2_O_2_ in 70% ethanol for 30 min, and blocking of non-specific binding with inactivated goat serum (diluted 1:5 in phosphate-buffered saline, PBS, pH 7.2). Primary antibodies were applied for 1 h at ambient temperature (for anti-CLDN antibodies) or overnight at 4 °C (for anti-CK antibodies) followed by incubation with biotinylated goat anti-mouse or goat anti-rabbit secondary antibodies (Vector Laboratories, Burlingame, CA, USA), respectively. Subsequently, avidin-biotin-peroxidase reagent (Vector Laboratories) was used according to the manufacturer’s instructions for 30 min and for anti-CLDN antibodies amplification was achieved using biotinylated tyramine as described elsewhere [44]. Supplemented with H_2_O_2_, 3,3′-diaminobenzidine (Sigma Aldrich, Munich, Germany) was applied to induce a brown colour reaction, and sections were counterstained with Mayer’s haematoxylin.

### 4.4. Immunofluorescence

Immunofluorescence was performed for cell cultures T120A, p.37–42; T124, p.15; DT14/04T, p.4 and p.16–19; and DT14/06T, p.44–49. The cells were cultivated on collagen coated cover slips. After achieving a confluency of about 75%, the cells were fixed with 4% formaldehyde for 10 min at room temperature and permeabilized with 0.3% Triton X-100 in PBS for 15 min at 37 °C. BSA (bovine serum albumin, 1%) in PBS was used for 30 min at 37 °C to block and saturate the non-specific binding positions. Then the cells were stained over night at 4 °C for CLDN-1, -3, -4 and -7 proteins with the respective primary antibodies according to Table 5. The secondary fluorescein conjugated anti-rabbit (Merck Millipore, Darmstadt, Germany) and fluorescein isothiocyanate (FITC) conjugated anti-mouse (Merck Millipore) were diluted 1:100 in PBS containing 1% BSA and added to the cells with 2 µM DAPI (Sigma-Aldrich, Munich, Germany) for 1 h at 37 °C. The cells were stored in PBS at 4 °C for further analysis. As a control for unspecific binding sites, cells were also incubated only with the secondary antibodies. The fluorescent images of the cells were collected with a Nikon Eclipse TE2000-E confocal laser scanning microscope (400 nm for Dapi and 488 nm for the CLDN proteins), with a 60× water immersion objective and EZ-C1 3.80 software program (Nikon, Düsseldorf, Germany). Image processing was performed using the ImageJ plugin named FigureJ [51].

## Figures and Tables

**Table 1:** This table shows the cell lines and primaray cultures and the corresponding histological classification (“Histological classif.”) of the original tissue samples. An “X” marks the tissues and cell pellets (“p.n”) that were available for gene expression analyses using QuantiGene Plex Assay.

**Cell Line**	**Histological Classif.**	**Tissue**	**p.00**	**p.01**	**p.02**	**p.03**	**p.05** *	**p.07** *	**p.08** *	**p.10**	**p.16**	**p.20**	**p.30**	**p.40**
DT14/05R	non-neoplastic mammary gland tissue	X		X						X		X		
DT14/06R		X	X							X		X	X	X
T124	lobular hyperplasia				X					X		X	X	
DT14/04T	simple adenoma	X				X				X		X	X	
T121	complex adenoma									X		X	X	
T120A	carcinoma, complex type									X		X	X	
DT14/06T		X	X							X		X	X	X
DT14/06Ts		X	X							X		X	X	
T126	carcinoma arising in a benign mixed tumour	X		X						X		X		
DT14/10	benign mixed mammary tumour				X					X		X	X	
DT15/02T					X					X		X	X	
**Primary Culture**	**Histological Classif.**	**Tissue**	**p.00**	**p.01**	**p.02**	**p.03** *	**p.05**	**p.07**	**p.08**	**p.10**	**p.16**	**p.20**	**p.30**	**p.40**
DT14/04R	non-neoplastic mammary gland tissue	X								X		**	**	**
DT14/05S2		X			X					X		**	**	**
DT14/07R			X	X				X				**	**	**
DT14/08R	lobular hyperplasia	X	X	X			X					**	**	**
DT14/07T	simple tubular carcinoma	X	X							X	X	**	**	**
DT14/09T		X	X	X					X			**	**	**

“*” p.05, 07 and 08 of cell lines had not been analysed, p.03 of primary cultures had not been analysed;“ **” p. > 19 of primary cultures do not exist, as primary cultures were designated as cell lines in case they reached p.19 and above.

**Table 2:** This table shows the cytokeratin (CK) distribution of the primary cultures. “Histological Classif.” refers to the histological classification of the original tissue samples from which the primary cultures were derived, “p.n” refers to a certain passage, “AE1/AE3” refers to Pan-CK (CK1-8, CK10, CK13-16, CK19), “MNF116” refers to Pan-CK (CK5, CK6, CK8, CK17, CK19), “HMW” refers to Pan-CK (CK1, CK5, CK10, CK14). “+” refers to positivity, “(+)” to a weak positivity and “−” refers to negativity for protein distribution. “NA” refers to not available.

Primary Culture	Histological Classif.	p.n	AE1/AE3	MNF116	HMW	CD5/6	CK7	CK10	CK14	CK20
DT14/04R	healthy mammary gland tissue	p.08	−							
		p.13	−							
DT14/05S2		p.04	−							
		later	NA							
DT14/07R		p.03	−	(+)	−				(+)	−
		p.07	+	+	−	+	+	−	+	−
DT14/08R	lobular hyperplasia	p.02	(+)	−						
		p.05	−							
DT14/07T	simple tubular carcinoma	p.03	+	+	+	+	(+)	−	+	−
		p.17	+	+	+	(+)	−		+	−
DT14/09T		p.03	+	(+)	(+)	−			(+)	−
		p.08	−							

**Table 3:** This table shows the cytokeratin (CK) distribution of the cell lines. “Histological Classif.” refers to the histological classification of the original tissue samples from which the cell lines were derived, “p.n” refers to a certain passage, “AE1/AE3” refers to Pan-CK (CK1-8, CK10, CK13-16, CK19), “MNF116” refers to Pan-CK (CK5, CK6, CK8, CK17, CK19), “HMW” refers to Pan-CK (CK1, CK5, CK10, CK14). “+” refers to positivity, “(+)” to a weak positivity and “−” to negativity for protein distribution. “NA” refers to “not available”.

Cell Line	Histological Classif.	p.n	AE1/AE3	MNF116	HMW	CK5/6	CK7	CK10	CK14	CK20
DT14/05R	healthy mammary gland tissue	p.04	(+)	−					(+)	−
		p.21	−							
DT14/06R		p.04	(+)	(+)	−				(+)	−
		p.41	−	NA	NA	NA	NA	NA	NA	NA
T124	lobular hyperplasia	p.03	+	+	+	+	(+)	(+)	+	−
		p.36	+	+	+	+	+	−	+	−
DT14/04T	simple adenoma	p.05	+	+	(+)	(+)	−		−	−
		p.27	+	+	−	+	+	−	+	−
T121	complex adenoma	p.06	(+)	(+)	(+)	(+)	−		+	−
		p.28	(+)	−	(+)	−			+	−
T120A	carcinoma complex type	p.07	(+)	(+)	(+)	(+)	(+)	−	+	−
		p.44	+	+	+	−			+	−
DT14/06T		p.04	(+)	−					(+)	−
		p.43	(+)	−						
DT14/06Ts		p.04	(+)	(+)	(+)	−			(+)	−
		p.33	(+)	−						
T126	carcinoma arising in a benign mixed tumour	p.03	−						(+)	−
		p.24	−							
DT14/10	benign mixed mammary tumour	p.02	+	+	NA	−	(+)	−	+	−
		p.31	+	+	NA	−			+	−
DT15/02T		p.04	+	(+)	+	−			+	−
		p.45	(+)	−	(+)	−			(+)	−

**Table 4:** This table shows the respective *CLDN-1*, *-3*, *-4* and *-7* gene expressions for cell lines T120A, T124, DT14/04T, DT14/04R, DT14/10, DT14/06T and T121 in early (“e.p.”) and late (“l.p.”) passages obtained by conventional PCR. “+” refers to positive expression, “−” refers to no expression.

Gene	*CLDN-1*		*CLDN-3*		*CLDN-4*		*CLDN-7*	
passage	e.p.	l.p.	e.p.	l.p.	e.p.	l.p.	e.p.	l.p.
T120A	+	+	+	+	+	+	+	+
T124	+	+	+	+	+	+	+	+
DT14/04T	+	+	+	+	+	+	+	+
DT14/04R	+	+	−	+	−	+	−	+
DT14/10	+	+	+	−	−	−	−	−
DT14/06T	−	+	−	−	−	−	−	−
T121	−	+	+	−	−	−	+	−

**Table 5:** List of antibodies used for immunocytochemistry (ICC) and immunofluorescence (IF). Staining pattern of all antibodies is identical to that described in the literature. “*” cross-reactivity with canine according to manufacturer’s information, “**” the canine KRT14 gene is a homolog of the human *KRT14* gene (National Center for Biotechnology Information HomoloGene). Cross reactivity of the antibody is highly presumable as amino acid sequences of the human (NP_00517.2) and canine (NP_001240670.1) cytokeratin 14 reveal an identity of 97% (according to National Center for Biotechnology Protein Blast).

Protein	Antibody	Dilution	
		ICC	IF
CLDN-1	Polyclonal rabbit anti-human CLDN-1 antibody (Thermo Fischer Scientific, Darmstadt, Germany) *	1:50	1:50
CLDN-3	Polyclonal rabbit anti-mouse CLDN-3 antibody (Thermo Fischer Scientific) *	1:200	1:83
CLDN-4	Monoclonal mouse anti-human CLDN-4 antibody (Clone 3E2C1, Thermo Fischer Scientific) *	1:200	1:83
CLDN-7	Polyclonal rabbit anti-human CLDN-7 antibody (Thermo Fischer Scientific) *	1:200	1:125
Pan-CK (CK1-8, CK10, CK13-16, CK19)	Monoclonal mouse anti-human CK (clone AE1/AE3, Dako, Hamburg, Germany) [45,46,47]	1:500	-
Pan-CK (CK5, CK6, CK8, CK17, CK19)	Monoclonal mouse anti-human CK (clone MNF116, Dako) [46] *	1:1000	-
Pan-CK (CK1, CK5, CK10, CK14)	Monoclonal mouse anti-human CK, high molecular weight (clone 34bE12, Dako) [48]	1:500	-
CK5 and CK6	Monoclonal mouse anti-human CK5/CK6 (clone D5/16B4, Dako) [49]	1:100	-
CK7	Monoclonal mouse anti-human CK7 (clone OV-TL12/30, Dako) [47]	1:100	-
CK10	Monoclonal mouse anti-human CK10 (clone DE-K10, Dako) [50] *	1:100	-
CK14	Polyclonal rabbit anti-human CK14 (Thermo Fischer Scientific) **	1:500	-
CK20	Monoclonal mouse anti-human CK20 (clone K_s_20.8, Dako) [47] *	1:100	-
